# Search for Nodulation and Nodule Development-Related Cystatin Genes in the Genome of Soybean (*Glycine max*)

**DOI:** 10.3389/fpls.2016.01595

**Published:** 2016-10-25

**Authors:** Songli Yuan, Rong Li, Lei Wang, Haifeng Chen, Chanjuan Zhang, Limiao Chen, Qingnan Hao, Zhihui Shan, Xiaojuan Zhang, Shuilian Chen, Zhonglu Yang, Dezhen Qiu, Xinan Zhou

**Affiliations:** ^1^Key Laboratory of Oil Crop Biology, Ministry of AgricultureWuhan, China; ^2^Oil Crops Research Institute of Chinese Academy of Agriculture SciencesWuhan, China; ^3^Bioinformatics Laboratory, College of Life Sciences, Xinyang Normal UniversityXinyang, China

**Keywords:** soybean, cystatin, genome-wide survey, gene expression, root nodule symbiosis

## Abstract

Nodulation, nodule development and senescence directly affects nitrogen fixation efficiency, and previous studies have shown that inhibition of some cysteine proteases delay nodule senescence, so their nature inhibitors, cystatin genes, are very important in nodulation, nodule development, and senescence. Although several cystatins are actively transcribed in soybean nodules, their exact roles and functional diversities in legume have not been well explored in genome-wide survey studies. In this report, we performed a genome-wide survey of cystatin family genes to explore their relationship to nodulation and nodule development in soybean and identified 20 cystatin genes that encode peptides with 97–245 amino acid residues, different isoelectric points (pI) and structure characteristics, and various putative plant regulatory elements in 3000 bp putative promoter fragments upstream of the 20 soybean cystatins in response to different abiotic/biotic stresses, hormone signals, and symbiosis signals. The expression profiles of these cystatin genes in soybean symbiosis with rhizobium strain *Bradyrhizobium japonicum strain 113-2* revealed that 7 cystatin family genes play different roles in nodulation as well as nodule development and senescence. However, these genes were not root nodule symbiosis (RNS)—specific and did not encode special clade cystatin protein with structures related to nodulation and nodule development. Besides, only two of these soybean cystatins were not upregulated in symbiosis after ABA treatment. The functional analysis showed that a candidate gene *Glyma.15G227500* (*GmCYS16*) was likely to play a positive role in soybean nodulation. Besides, evolutionary relationships analysis divided the cystatin genes from *Arabidopsis thaliana, Nicotiana tabacum*, rice, barley and four legume plants into three groups. Interestingly, Group A cystatins are special in legume plants, but only include one of the above-mentioned 7 cystatin genes related to nodulation and nodule development. Overall, our results provide useful information or clues for our understanding of the functional diversity of legume cystatin family proteins in soybean nodulation and nodule development and for finding nodule-specific cysteine proteases in soybean.

## Introduction

Nitrogen fixation efficiency plays important roles in plant cultivation and fertilizer application and is closely related to nodulation, nodule development, and senescence (Biswas and Gresshoff, 2014). Previous studies have shown that cysteine proteases are important in nodule development and senescence in several legume plants. For example, silencing of AsNODF32 delays root nodule development and bacteroid senescence and prolongs nodule lifespan in *Astragalus sinicus* (Li et al., 2008). Inhibition of cysteine protease CYP15A delays nodule senescence in *Medicago trunctula* (Sheokand et al., 2005) and protease PsCyp15A is activated at the onset of senescence in the indeterminate nodules of pea (Vincent and Brewin, 2000). In soybean, since the first report of cysteine protease expression during nodule senescence in 1983 (Pfeiffer et al., 1983), 18 soybean cysteine proteases have been found actively transcribed during nodule development and senescence (van Wyk et al., 2014). However, it is not well understood whether these cysteine proteases play specific roles in nodule development and senescence and why so many soybean cysteine proteases are involved in regulation of nodule symbiosis. Studies on their nature inhibitors could help us better understand these questions.

Cystatins are a group of small proteins known to reversibly inhibit the activity of cysteine proteases in families of papain C1A and legumain C13 peptidase (Martinez et al., 2007). Their inhibitory mechanism involves a tripartite wedge formed by the partially flexible N terminus containing a Gly residue and two hairpin loops that carry a conserved QxVxG motif and a conserved tryptophan (Trp) residue (Stubbs et al., 1990). Besides, a conserved LARFAV motif ([LVI]-[AGT]-[RKE]-[FY]-[AS]-[VI]) is also common to plant cystatins (Margis et al., 1998). Disturbance of the equilibrium between cystatins and their cysteine proteases in plant is a key event in endogenous protein turnover (Martinez et al., 2009), accumulation and mobilization of storage proteins (Benchabane et al., 2010), seed germination and maturation (Arai et al., 2002), programmed cell death (Belenghi et al., 2003), abiotic environmental stresses (Hwang et al., 2010), and protection of plants against attack by mites (Carrillo et al., 2011), fungi (Martinez et al., 2003; Popovic et al., 2013), and viruses (Gutierrez-Campos et al., 1999). Although previous studies have well described the genome-wide characteristics of cystatin family genes in four different species, *Hordeum vulgare* (Martinez et al., 2009), *Nicotiana tabacum* (Zhao et al., 2014), *Apple* (Tan et al., 2014), and *Oryza sativa* L. (Wang et al., 2015), the genome-wide studies of cystatin family genes in legume are quite limited.

Soybean (*Glycine max*) is an important oil crop, food and feed material with planting area >1734 million acres worldwide in 2015. Twenty soybean cystatins have already been identified by releasing of the complete soybean genome sequence (Schmutz et al., 2010) and the RNA-seq atlas showed they were expressed in 14 different soybean tissues, especially in nodules (Severin et al., 2010). In addition, the inhibitory activities of these cystatins were analyzed and seven of them were actively transcribed in nodules (van Wyk et al., 2014). Ectopic expression of rice phytocystatin positively regulates nodulation and nitrogen deficiency of soybean (Quain et al., 2015). However, almost all of these studies were focused on individual cystatin members. Which soybean cystatins play roles in symbiotic signal transduction, nodulation, nodule development, and senescence is still incompletely understood.

In this report, the detail information of genome-wide identification and characterization of 20 soybean cystatins were shown to reveal the special characteristics of soybean cystatin family genes. Expression profiles of cystatin genes in soybean symbiosis with *Bradyrhizobium japonicum* strain 113-2 were used to explore their putative roles in nodulation and nodule development. And the function analysis of *Glyma.15G227500* (*GmCYS16*) was used to confirm the expression of these soybean cystatin genes in symbiosis. These investigations and analyses could provide useful information for the research of the functional diversity of legume cystatin family proteins, and helpful clues for investigation of nodule-specific cysteine proteases in soybean.

## Materials and methods

### Database searches for the identification of cystatin family genes from soybean, *L. japonicus, M. truncatula*, common bean, *A. thaliana, N. tabacum*, rice and barely

To identify cystatin family genes in soybean, the Soybean Genome Database (http://soybase.org/), Phytozome Database (http://www.phytozome.net/soybean), and NCBI-BLAST (http://blast.ncbi.nlm.nih.gov/) online resources were searched to identify the entire family of cystatins in *G. max*. Soybean cystatins were applied as model for identification of cystatin homologs in *Lotus japonicus, Medicago truncatula*, common bean, *Arabidopsis thaliana*, rice and barely at LIS (Legume Information System) (http://legumeinfo.org/lis_gene_families/chado/msa/phytozome_10_2.59086800-consensus/). *N. tabacum* cystatin proteins were identified in NCBI-BLAST (http://blast.ncbi.nlm.nih.gov/) online resources.

### Identification of conserved motifs and phylogenetic tree constructions

Multiple alignments of amino acid sequences of soybean cystatin family genes were performed with DNAMAN software to retrieve the conserved motifs of soybean cystatins. Phylogenetic tree analysis was performed using MEGA6 software (Tamura et al., 2013). Multiple alignments of the full-length deduced amino acid sequences of cystatin family genes were conducted with Clustal W program. The Poisson substitution model, uniform rates, pairwise deletion and 1000 Bootstrapping replicates were applied in Neighbor-joining method to perform phylogenetic tree analysis.

The different cystatins including 20 cystatin proteins from soybean, 34 cystatin proteins from *M. trunctula*, 7 cystatin proteins from *L. japonicus*, 10 cystatin proteins from common bean, 7 cystatin proteins from *A. thaliana*, 10 cystatin proteins from *N. tabacum*, 18 cystatin proteins from rice, and 13 cystatin proteins from barley were applied for phylogenetic and molecular evolutionary analyses using the programs RAxML and MEGA version 6.0 (http://www.megasoftware.net; Guindon and Gascuel, 2003; Tamura et al., 2013). Maximum likelihood (ML) analysis was performed using RAxML 8.0.0 (Stamatakis, 2014) and rapid bootstrap analysis was performed with the bootstrap convergence test using the extended majority-rule consensus tree criterion (autoMRE) in RAxML. GenBank accession numbers are listed in Table S1.

### Soybean cystatin genes sequence and structure analysis

The genomic DNA and cDNA sequences corresponding to each predicted soybean cystatin genes were downloaded from Phytozome Database (http://www.phytozome.net/soybean) and Soybean Genome Database (http://soybase.org/). The gene structure display server program (http://gsds.cbi.pku.edu.cn) (Guo et al., 2007) was used to analyze the exon/intron structures of these 20 soybean cystatin genes. The SignalP version 3.0 program (http://www.cbs.dtu.dk/services/SignalP) (Bendtsen et al., 2004) was used to perform the signal peptide analysis. The secondary structures (a-helix and b-sheets) were predicted as previously reported (http://ps2.life.nctu.edu.tw/) (Chen et al., 2009). The automated SWISS-MODEL program (http://swissmodel.expasy.org/interactive) (Peitsch, 1996) was used to model the three-dimensional structures of soybean cystatins, and the known crystal structure of rice oryzacystatin I (OC-I) (Nagata et al., 2000), SiCYS (Hu et al., 2015), and PMC (Green et al., 2013) were used to construct the homology-based models. The Plant CARE database (http://bioinformatics.psb. ugent.be/ webtools/ plantcare/html/) (Lescot et al., 2002) was used to analyze the promoter sequences of soybean cystatins.

### Plant materials and treatments

Seeds of soybean Tianlong No.1 (stored in our lab) were surface-sterilized, germinated and grown on moistened filter paper in a greenhouse with a 16/8 h day/night cycle and 70% relative humidity (RH) for 2–3 d at 28°C (Yuan et al., 2016). For nodulation experiments, the germinated soybean seeds were grown in pots filled with sterilized vermiculite and sand (1:1) supplemented with half-strength B&D medium in a chamber with a 16/8 h day/night cycle at 28°C for 4–5 d before inoculation with rhizobium strain *113-2* (stored in our lab). After inoculation, plants were kept under the same growth conditions.

For RNA isolation, soybean roots from CK and *113-2* inoculated groups were collected with three biological replicates at five different time points [0.5 h, 7–24 h (mixture of 7 h and 24 h), 5, 16, and 21 d of post inoculation] (Yuan et al., 2016) and frozen at −80°C, and soybean nodules from CK and *113-2* inoculated groups were collected with three biological replicates at five important nodule development time points (12, 30, 42, 60, and 84 d of post inoculation) and frozen at −80°C.

For ABA treatment, nodules-containing, strain *113-2* inoculated soybean roots were collected at 6, 33, and 62 d of post inoculation, transferred into containers with half-strength B&D medium supplemented with 200 μM ABA, and cultured for 5, 10, and 9 d, respectively. The collected soybean roots samples were collected with three biological replicates and were frozen at −80°C for RNA isolation.

### RNA extraction, semi-quantitative RT-PCR, and qPCR

Total RNA was isolated using TRIzol reagent (Invitrogen, USA) and stored in a −80°C freezer for downstream gene-expression experiments. Potential genomic DNA were removed using RNeasy plant mini kit (QIAGEN, Germany) and RNA quantity and quality were measured using an Epoch Multi-Volume Spectrophotometer system, NanoDrop and Agilent 2100 Bioanalyzer (Agilent Technologies, Palo Alto, CA, USA). *Glyma.15G227500* expression in *L. japonicus* were detected using semi-quantitative RT-PCR at the following conditions: an initial denaturation at 94°C for 5 min, followed by 31 cycles of 30 s at 94°C, 30 s at 55°C, and 30 s at 72°C, as well as a final extension at 72°C for 10 min. PCR products were detected by 1% agarose gel analysis. Expressions of other genes in *L. japonicus* were determined with qPCR using primer sets listed in Table S2, and following cycling conditions: 30 s at 95°C, followed by 40 cycles of 5 s at 95°C, 15 s at 60°C and 12 s at 72°C and final 5 s at 72°C. The soybean QACT and *L. japonicus* polyubiquitin transcript were used as the internal, endogenous control to normalize the samples, and the 2^−ΔΔCT^ method (Livak and Schmittgen, 2001) was used to analyze the relative changes in gene expression in qPCR experiments. Three biological replica samples were used to detect the expression of soybean cystatins in symbiosis with *B. japonicum* strain 113-2, and three or four replicate reactions per sample were used to ensure statistical credibility. Student's *t*-test was conducted to analyze the differences in qPCR experiments using software SPSS Statistics 17.0.

### Overexpression of *Glyma.15G227500* in *L. japonicus* by hairy root transformation

The full-length coding sequence of *Glyma.15G227500* was cloned into the *KpnI/ BamHI* site of pU1301 using primer set of 5′-AGGGTACCATGAGAGCATTAACC TCTTC-3′ and 5′-CAGGATCCTTAGGAATGATCTTGTTCC-3′ to generate pMUb: Glyma.15G227500. *A. rhizogenes* strain LBA1334 cells carrying pMUb: Glyma.15G227500 were used to induce hairy root formation in wild-type *L. japonicus* “MG-20” using an *A. rhizogenes*-mediated procedure as described previously (Yuan et al., 2012). Briefly, seedlings were cut at the base of the hypocotyls, placed in suspension of *A. rhizogenes* LBA1334 cells containing plasmids for 30 min, and transferred onto agar plates of Murashige and Skoog (MS; Sigma) medium containing 1.5% (w/v) Suc and cocultivated for 5 d. The plants were then transferred onto agar plates containing 250 mg/ml of cefotaxime and grown until hairy roots (a few cm long) developed from the base of hypocotyls. Each hairy root was properly labeled and a short root tip (1- to 3-mm long) was removed to test for GUS activity in staining solution overnight at 37°C in the dark. Hairy roots with GUS-positive tips were preserved and allowed to continue to grow. Each seedling was allowed to have 1–2 transgenic hairy roots. Plants with transgenic hairy roots were transferred to pots filled one-half-strength Broughton&Dilworth (B&D) medium supplemented with vermiculite and sand (1:1) (Broughton and Dilworth, 1971) and grown in a chamber in a 16-/8-h day/night cycle at 23°C. After a week of adaptation, plants were inoculated with *Mesorhizobium loti* strain MAFF303099 and grown in the same medium without ammonium nitrate. Nodulation phenotypes of the transgenic hairy roots were scored at 32 days of post inoculation with *M. loti*. The transgenic hairy roots expressing the empty vector (pU1301) were used as the control.

## Results

### Characterization of the soybean cystatin genes

The Soybean Genome Databases were searched to identify the entire family of cystatins in *G. max*. Twenty full-length soybean cystatin cDNAs containing complete ORF were obtained and named as GmCYS1- GmCYS20 according to their positions in chromosomes. Table 1 lists their detailed information. The identified soybean cystatin genes encode peptides with 97–245 amino acid residues and isoelectric point (pI) of 4.98–9.88. SignalIP was used to find signal peptides. Among the 20 soybean cystatins, 14 soybean cystatins have signal peptides of 17–33 amino acid residues and the other six cystatins (GmCYS9, GmCYS12, GmCYS13, GmCYS14, GmCYS18, and GmCYS20) has no signal peptide. The cDNA sequences of each cystatin were compared with their genomic sequences to analyze their exon/intron structures. The results revealed that 12 soybean cystatins are single-exon cystatins without intron, 3 soybean cystatins (GmCYS9, GmCYS12, and GmCYS20) contain one intron, 3 (GmCYS13, GmCYS14, and GmCYS17) contain two introns and 2 (GmCYS10 and GmCYS16) contain three introns. The secondary structures (α-helix and β-strand) of cystatin genes were predicted in http://ps2.life.nctu.edu.tw/, and the results are shown in Table 1. Among the 20 soybean cystatins, 14 have two α-helixes, 3 (GmCYS9, GmCYS12, and GmCYS18) have only one α-helix, one (GmCYS14) has three α-helixes, and 2 (GmCYS10 and GmCYS16) have six α-helixes. The number of β-strand is divergent from 4 to 11 in these 20 cystatins.

**Table 1:** **Detailed information of soybean cystatin family genes**.

**Name**	**Soybean gene ID**	**Chromosome location**	**No. of amino acid residues**	**PI**	**Signal peptide**	**α-Helix**	**β-Strand**	**Exon**	**Intron**
GmCYS1	Glyma.04G096400	Chr04 8659349–8659856	114	9.88	17	2	4	1	0
GmCYS2	Glyma.05G149800	Chr05 34385547–34386483	130	9.23	26	2	4	1	0
GmCYS3	Glyma.07G266000	Chr07 43976589–43977195	114	7.77	20	2	4	1	0
GmCYS4	Glyma.08G168600	Chr08 13381885–13382591	125	9.41	19	2	6	1	0
GmCYS5	Glyma.09G010900	Chr09 837176–837835	114	9.88	17	2	4	1	0
GmCYS6	Glyma.09G110600	Chr09 21896029–21896583	114	9.88	17	2	4	1	0
GmCYS7	Glyma.11G064200	Chr11 4842312–4842656	114	9.83	17	2	4	1	0
GmCYS8	Glyma.11G253300	Chr11 34413583–34414431	126	9.52	20	2	4	1	0
GmCYS9	Glyma.13G071800	Chr13 17264906–17266886	97	5.83		1	6	2	1
GmCYS10	Glyma.13G189500	Chr13 30296749–30300668	245	6.56	33	6	11	4	3
GmCYS11	Glyma.13G209000	Chr13 32301762–32302169	124	5.73	19	2	4	1	0
GmCYS12	Glyma.14G038200	Chr14 2854471–2855966	103	5.8		1	5	2	1
GmCYS13	Glyma.14G038300	Chr14 2859830–2861347	200	5.26		2	9	3	2
GmCYS14	Glyma.14G038500	Chr14 2876239–2877988	184	4.98		3	9	3	2
GmCYS15	Glyma.15G115300	Chr15 9076848–9077830	112	9.07	17	2	5	1	0
GmCYS16	Glyma.15G227500	Chr15 42044582–42049151	245	7.27	33	6	9	4	3
GmCYS17	Glyma.18G003700	Chr18 304856–305625	142	9.56	20	2	4	2	2
GmCYS18	Glyma.18G103700	Chr18 11309282–11311702	101	5.64		1	5	1	0
GmCYS19	Glyma.19G206500	Chr19 46202432–46203385	114	9.88	17	2	4	1	0
GmCYS20	Glyma.20G045500	Chr20 8475315–8478532	97	5.83		2	6	2	1

### Phylogenetic analysis and structural predication of the soybean cystatins

**Figure 1:**
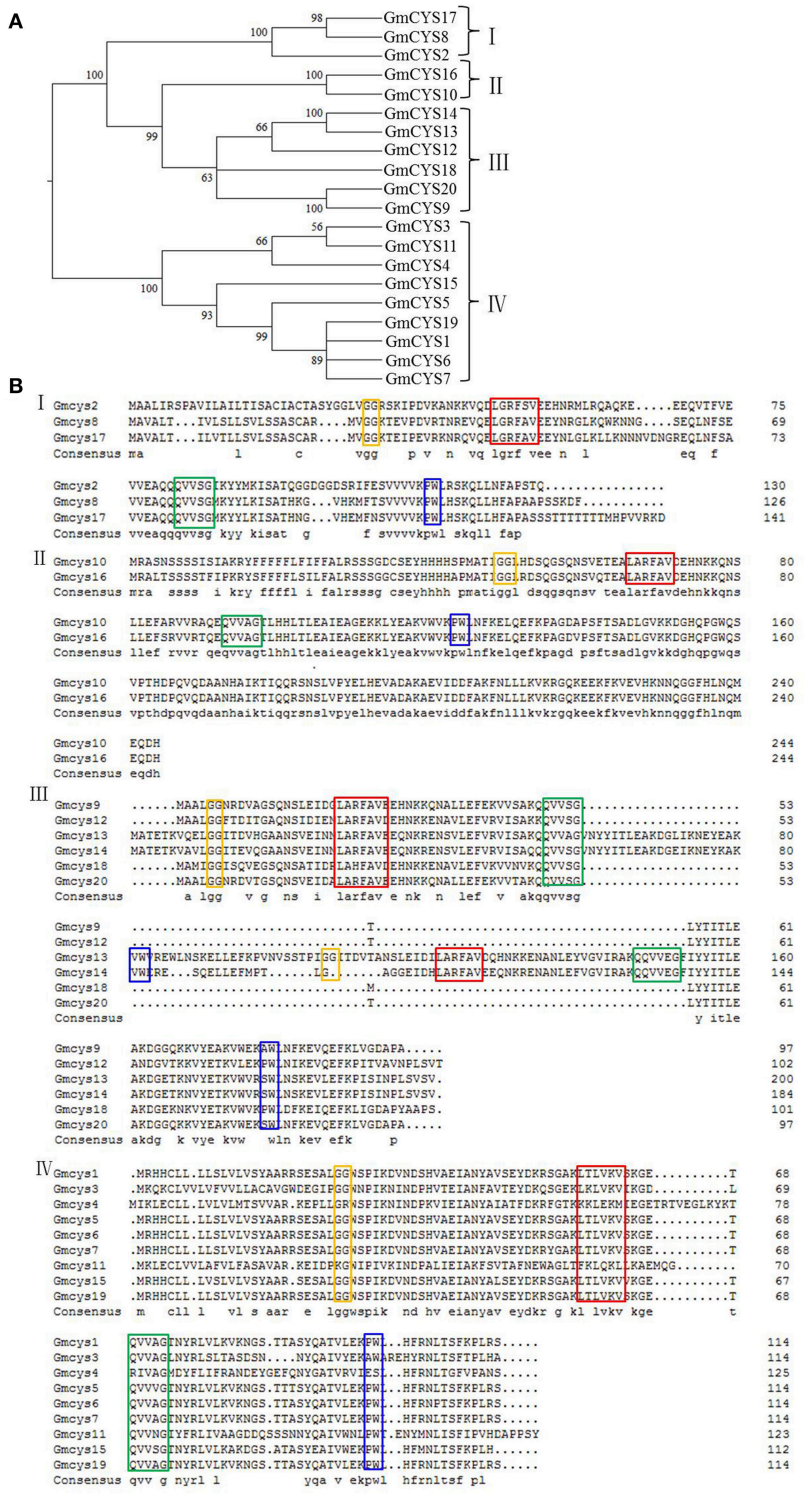
**Phylogenetic analysis and amino acid residue alignment of soybean cystatins. (A)** Phylogenetic analysis of soybean cystatins. Phylogenetic tree construction of soybean cystatins is based on the full-length deduced amino acid sequences using MEGA 6 by the neighbor-joining method with 1000 bootstrap replicates. The tree shows four major phylogenetic clades (clades I to IV) indicated with braces. **(B)** Alignments of conserved motifs of different clades of the soybean cystatins. The main cystatin conserved motifs in four phylogenetic clades (clades I to IV) are in orange, red, green, and blue boxes.

A phylogenetic analysis based on alignments of the 20 full-length cystatin protein sequences was performed to determine the phylogenetic relationships among the different members of the soybean cystatins, and the results are revealed in Figure 1A.

Twenty soybean cystatins were divided into four clades (I, II, III, and IV) in the neighbor-joining phylogenetic tree, and the members within each clade show similar gene structures (Table 1).

Alignments of cystatin sequences in different soybean clades were used to search for amino acid variants that could affect their inhibitory capability on cysteine proteases. The results are shown in Figure 1B. All protein signatures responsible for the cysteine proteinase inhibitory properties were conserved along the sequence of Clades I, II, and III, with exception of the alanine in the second position of the conserved LARFAV motif (A/G) in Clade I. Clade IV comprises 9 members (the largest number of members) with different amino acid residues in the conserved LARFAV motif. Besides, Clade III contains variable numbers of the four conserved motifs (Figure 1B). In addition, the sequence length of soybean cystatins in different clades also varies (Figure 1B).

**Figure 2:**
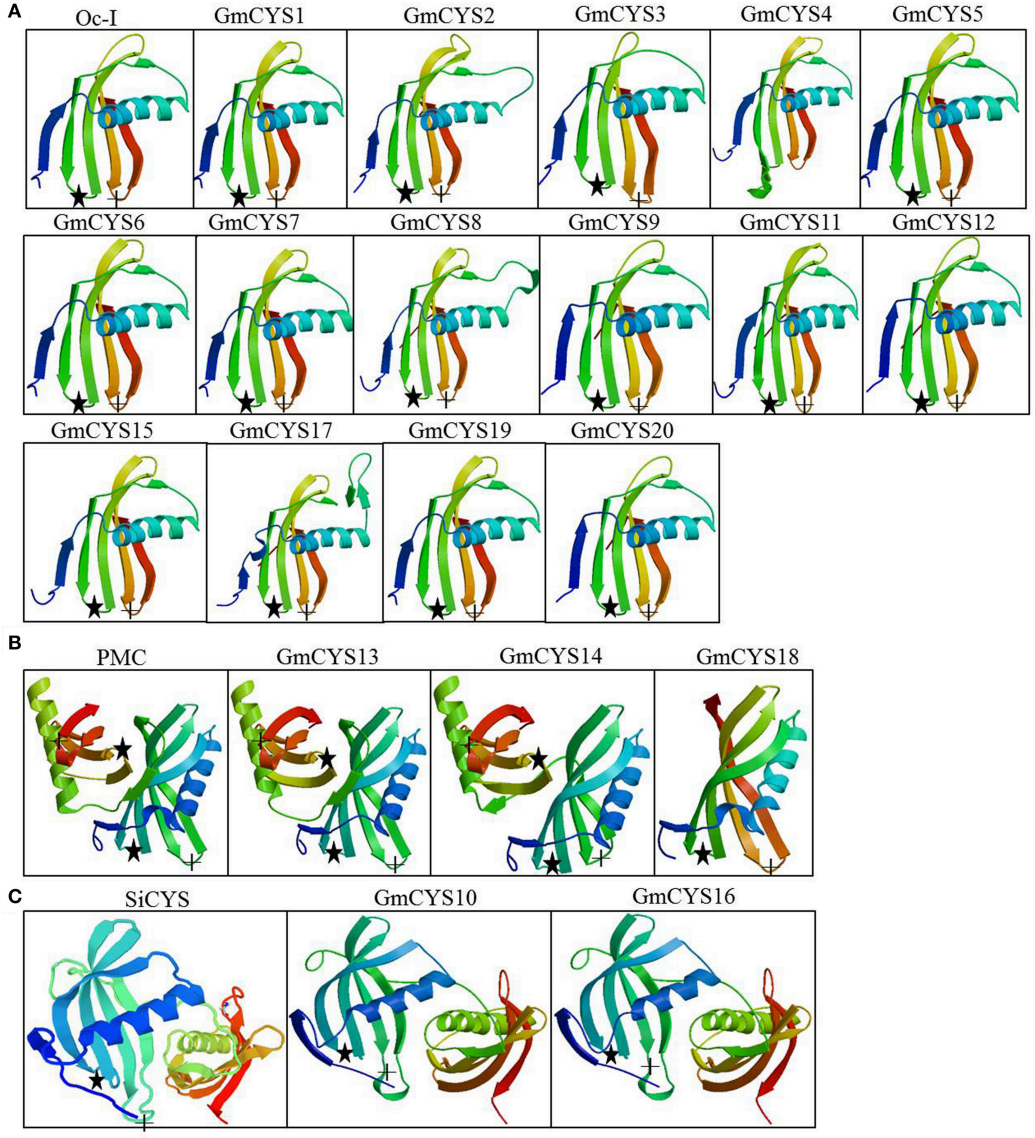
**The three-dimensional structure prediction of soybean cystatins. (A)** The three-dimensional structures of 15 cystatins were predicted using the automated SWISS-MODEL program with OC-I as a template. **(B)** The three-dimensional structures of GmCYS13, GmCYS14, and GmCYS18 were predicted using the automated SWISS-MODEL program with PMC as a template. **(C)** The three-dimensional structures of GmCYS10 and GmCYS16 were predicted using the automated SWISS-MODEL program with SiCYS as a template. Two important motifs involved in the interaction with the target enzymes are indicated the reactive site (asterisks) and W residue (crosses).

The predicted three-dimensional structures of the soybean cystatins were established using the automated SWISS-MODEL program with the known crystal structure of OC-I (Nagata et al., 2000), SiCYS (Hu et al., 2015), and PMC (Green et al., 2013) as templates (Figure 2). Although these structures were predicted with variable degrees of accuracy, GmCYS1 to GmCYS9, GmCYS11, GmCYS12, GmCYS15, GmCYS17, GmCYS19, and GmCYS20 share similar protein structures with rice OC-I (Figure 2A) and GmCYS13, GmCYS14 and GmCYS18 share similar protein structure with PMC (Figure 2B). Besides, GmCYS10 and GmCYS16 show significant variations in their predicted three-dimensional structures, and share similar protein structure with SiCYS (Figure 2C). Two important motifs (the conserved QxVxG motif and the conserved tryptophan) involved in the interaction with the cysteine proteinases were shown in Figure 2. The predicted structure of GmCYS4 shows some distortions in the region of QxVxG motif and W residue, probably due to the glutamine in the first position of the conserved QxVxG motif (R/Q) and change of the conserved tryptophan to phenylalanine (W/F) in this cystatin (Figure 1B).

### Evolutionary relationships among the cystatin family in rice, barley, *Arabidopsis thaliana, Nicotiana tabacum* and four legume plants

**Figure 3:**
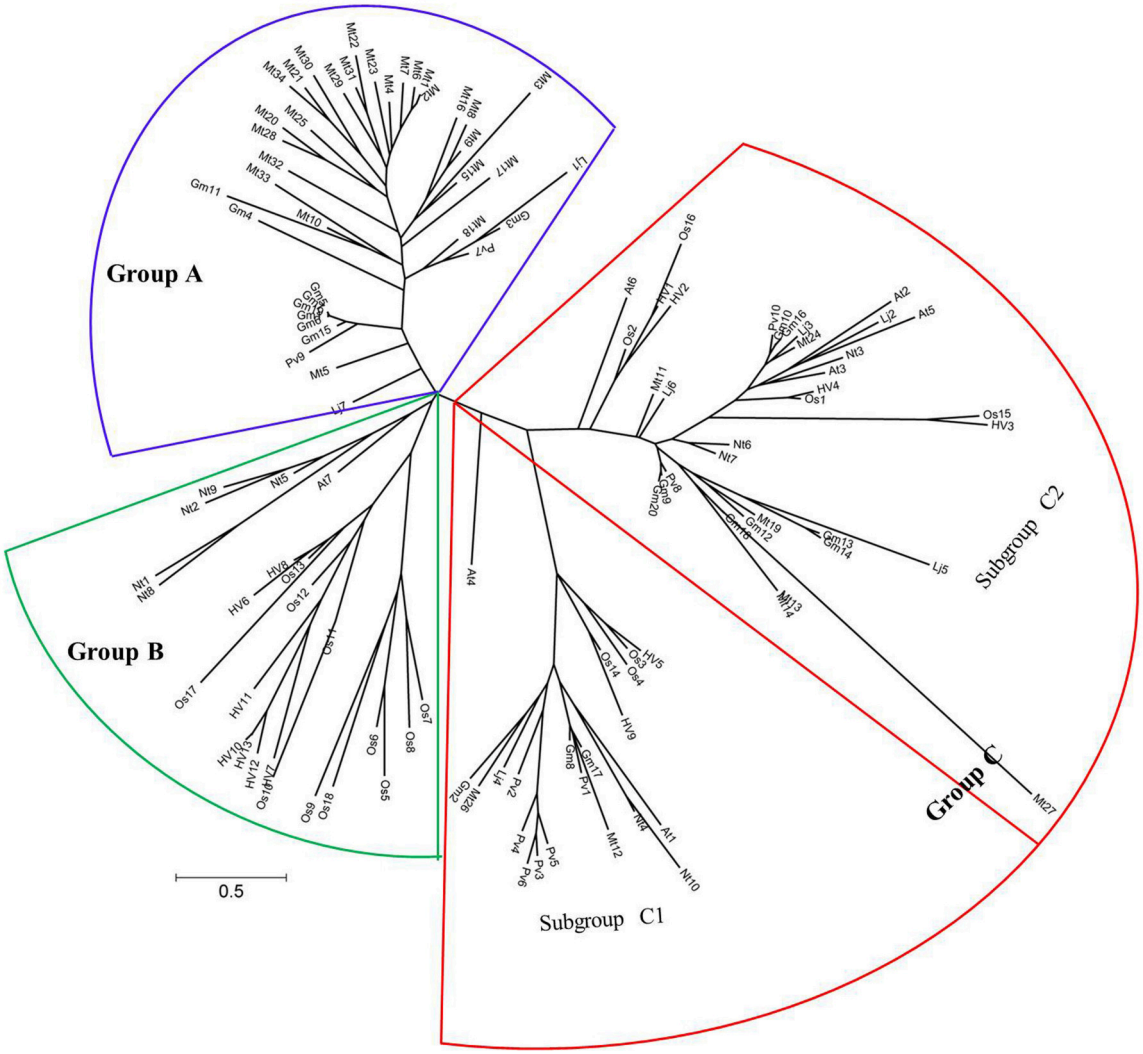
**Phylogenetic tree of cystatin proteins from soybean, ***M. trunctula***, ***L. japonicus***, common bean, ***A. thaliana***, ***N. tabacum***, rice and barley**. Twenty cystatin proteins from soybean, 34 cystatin proteins from *M. trunctula*, 7 cystatin proteins from *L. japonicus*, 10 cystatin proteins from common bean, 7 cystatin proteins from *A. thaliana*, 10 cystatin proteins from *N. tabacum*, 18 cystatin proteins from rice, and 13 cystatin proteins from barley were used to construct the phylogenetic tree using the programs RAxML and MEGA6.0. These cystatin proteins were classified into three groups, namely Group A, Group B, and Group C. Group C was subdivided in two subgroups: subgroup C1 and subgroup C2. GenBank accession numbers of these cystatin proteins are listed in Table S1.

To estimate the phylogenetic relationships of cystatins in legume plants to other known cystatins in rice, barley, *A. thaliana* and *N. tabacum*, a multiple sequence alignment of soybean cystatin protein sequences to the sequences from other three legume plants (*M. trunctula, L. japonicus*, and common bean) and four non-legume plants (*A. thaliana, N. tabacum*, rice, and barley) was conducted to establish the phylogenetic tree using the programs RAxML and MEGA version 6.0 (Figure 3). The accession numbers of cystatins in *M. trunctula, L. japonicus*, common bean, *A. thaliana, N. tabacum*, rice, and barley are shown in Table S1. These cystatin proteins from eight different species are distributed in three major groups (Group A, Group B, and Group C). Among them, Group A was formed by 39 cystatins with only legume plant cystatin, 26 *M. trunctula* cystatins, 9 soybean cystatins, 2 *L. japonicus* cystatins, and 2 common bean cystatin. Group B was the smallest group composed of 24 cystatins with only non-legume plant cystatin, 11 rice cystatins, 7 barley cystatins, 5 *N. tabacum* cystatins, and 1 *A. thaliana* cystatin. Group C was the largest group composed of 56 cystatins with 32 legume plant cystatins and 24 non-legume plant cystatins, and was subdivided in two subgroups: subgroup C1 and subgroup C2. Cystatins from soybean almost equally fall into Group A and Group C. Cystatins from *M. trunctula* mainly fall into Group A, and cystatins from common bean mainly fall into subgroup C1, while most cystatins from of *L. japonicus* are in subgroup C2. The phylogenetic relationship may reflect some distinction between legume plant cystatins and four non-legume plants cystatins (2 monocotyledon plants and 2 non-legume dicotyledonous plants), and indicate that the potential biological functions of some cystatins are conservative in legume plants and in some special developmental processes.

### Promoter sequence analysis of the soybean cystatins

Based on soybean Genome Database (http://www.phytozome.net/soybean), 3000 bp putative promoter fragments upstream of the soybean cystatins were obtained and putative cis-acting elements were analyzed with the Plant CARE database (http://bioinformatics.psb.ugent.be/ webtools/ plantcare/html/). Various putative plant regulatory elements in these promoters are shown in Table 2, including elements in response to environmental cues and hormone signals, such as temperature responsive elements (HSE and LTR), drought-inducibility elements (MBS), defense and stress responsiveness elements (TC-rich repeats), wound-responsive element (WUN-motif), fungal elicitor responsive element (Box-W1), circadian-regulated elements (circadian) and elements in response to hormones including abscisic acid (ABRE and CE3), gibberellin acid (GARE-motif, P-box, and TATC-box), salicylic acid (TCA-element), auxin (TGA-element, TGA-box, and AuxRR-core) and jasmonic acid (TGACG-motif), and 7 other putative regulatory elements including flavonoid biosynthetic genes regulation elements (MBSI and MBSII), nodule specific factor binding site element (Nodule-site2), MYBHv1 binding site element (CCAAT-box), anaerobic induction element (ARE), ethylene-responsive element (ERE), and elicitor-responsive element (ELI-box3). The statistical analyses of data in Table 2 showed that 95% soybean cystatins have TC-rich repeats element, 90% have HSE element, TGACG-motif and circadian elements, 80% have TCA-element, ABRE element and gibberellin acid elements, and so on. These results indicated that the expressions of these 20 soybean cystatins might be regulated by various environmental stresses and hormone signals.

**Table 2:** **Distribution of relative elements in upstream 3000 bp sequences of soybean CYS genes**.

**Gene CYS**	**1**	**2**	**3**	**4**	**5**	**6**	**7**	**8**	**9**	**10**	**11**	**12**	**13**	**14**	**15**	**16**	**17**	**18**	**19**	**20**
ABRE	4	2		3		2	1	2	2	3		4	1	1	3	2	3		1	2
ARE	1	1		2				4	4	4		2	3	3	4	3	4	3	1	6
GARE-motif	2		5	3	1		2	1	1		1	3			1		2		1	
P-box		1	1	2	1				1		2	1	1	1		3	1			
HSE	2	3	2	1	2	2	3	2		5	7	3	2	2	4	1		2	3	7
LTR	1	1				1		1							1		1			
TC-rich repeats	4	3	7	2	3	3	3	2	2	5	2		6	4	3	4	5	1	5	5
TGA-element	1	1	1	1	1	1		1		1	1							1	1	
TGACG-motif	3	2	3	3	3	3	4	2	7	3		3	1	1		1	1	2	3	4
Circadian	4	5	5	1	1	3	5	5	6	2		4	4	4		4	2	1	3	4
TCA-element		1		1		1	1	3	2	3		4	1	1	2	1	5	2	3	5
MBS		6	2	2	1	3	1	3	3		1	1	4				1		2	1
MBSI						1														
MBSII																		1		
Nodule-site2		1																		
Box-W1		1			1	1	1		2					1	2		2	1	1	
ERE				1		1				3	3				1		1			3
CCAAT-box	1	2		1		1			1	1										
WUN-motif				1		1		1					2	2	1		2			
AuxRR-core							1											1		
TATC-box								1	1			1					1			
CE3									1			1								
ELI-box3																		1		1
TGA-box																				1

### Expression profiles of soybean cystatin genes in symbiosis with *B. japonicum* strain *113-2*

Based on plant Phytozome database (http://www.phytozome.net/soybean), the expression profiles of the 20 soybean cystatins were investigated in various tissues, including nodules. symbiotic condition, root. symbiotic condition, flower, leaves, nodules, pod, root, root hairs, seed, and stem. The results showed that the soybean cystatin genes were expressed in distinct patterns and most of them were expressed in multiple tissues (Table 3). Among them, 10 soybean cystatins (*GmCYS2, GmCYS3, GmCYS9, GmCYS10, GmCYS12, GmCYS15, GmCYS16, GmCYS17, GmCYS18*, and *GmCYS20*) showed relative high expression in nodules and/or nodules at symbiotic condition, while other 6 soybean cystatins (*GmCYS1, GmCYS4, GmCYS5, GmCYS6, GmCYS7*, and *GmCYS19*) showed no expression in these two tissues. *GmCYS8* had no expression in nodules and very low expression in nodules at symbiotic condition while *GmCYS11* was not expressed in nodules at symbiotic condition, but had very low expression in nodules. In addition, 13 soybean cystatins (*GmCYS2, GmCYS3, GmCYS8, GmCYS9, GmCYS10, GmCYS12, GmCYS13, GmCYS14, GmCYS15, GmCYS16, GmCYS17, GmCYS18*, and *GmCYS20*) were expressed in root, root hairs and roots at symbiotic condition, and *GmCYS5* had low expression in roots at symbiotic condition. *GmCYS1, GmCYS5, GmCYS6, GmCYS7*, and *GmCYS19* were mainly expressed in flower, and *GmCYS4* was mainly expressed in pod and seed.

**Table 3:** **Expression analysis of 20 soybean cystatins in different tissues in phytozome database**.

**PKM (Phytozome database)**										
**Tissue**	**Flower**	**Leaves**	**Nodules**	**Pod**	**Root**	**Root Hairs**	**Seed**	**Stem**	**Nodules. symbiotic condition**	**Root. symbiotic condition**
GmCYS1	2.223	0	0	0.36	0	0	0.328	0	0	0
GmCYS2	65.448	5.741	14.347	9.69	7.617	14.376	299.668	2.31	106.506	104.914
GmCYS3	3.529	5.776	40.961	50.279	16.214	29.289	90.18	3.426	3.518	0.701
GmCYS4	0.261	0	0	31.197	0	0	78.61	0	0	0
GmCYS5	11.236	0	0	1.629	0	0	0.818	0	0	0.626
GmCYS6	1.973	0	0	0.107	0	0	0.041	0	0	0
GmCYS7	0.261	0	0	0.17	0	0	0.04	0	0	0
GmCYS8	0	0	0	0	3.17	2.938	243.649	0	0.048	0.173
GmCYS9	49.973	62.288	46.363	112.498	72.678	45.571	88.956	101.283	13.656	31.935
GmCYS10	98.534	57.723	55.677	65.267	95.346	71.81	229.767	63.73	31.794	30.9
GmCYS11	0	0	0.366	0.436	0	0	0.065	0	0	0
GmCYS12	215.075	67.664	9.519	500.369	41.927	25.159	62.095	95.536	0.487	8.882
GmCYS13	24.323	3.608	0.001	14.707	9.976	0.063	4.731	4.187	0.023	0.185
GmCYS14	312.627	24.379	0.289	323.377	24.549	1.237	1.185	55.414	0.005	0.109
GmCYS15	82.558	67.571	145.753	50.291	91.941	140.95	67.492	213.426	718.33	897.133
GmCYS16	45.481	21.699	19.798	35.242	32.294	24.917	48.012	29.017	16.074	21.96
GmCYS17	1.125	0	0	0.018	7.901	4.054	2.032	0	38.085	15.773
GmCYS18	0	0.032	3.491	0	26.299	3.067	0	0	40.862	205.927
GmCYS19	2.639	0	0	0.09	0	0	0.086	0	0	0
GmCYS20	94.949	71.153	64.095	111.068	73.98	57.858	192.429	108.603	13.033	8.785

**Figure 4:**
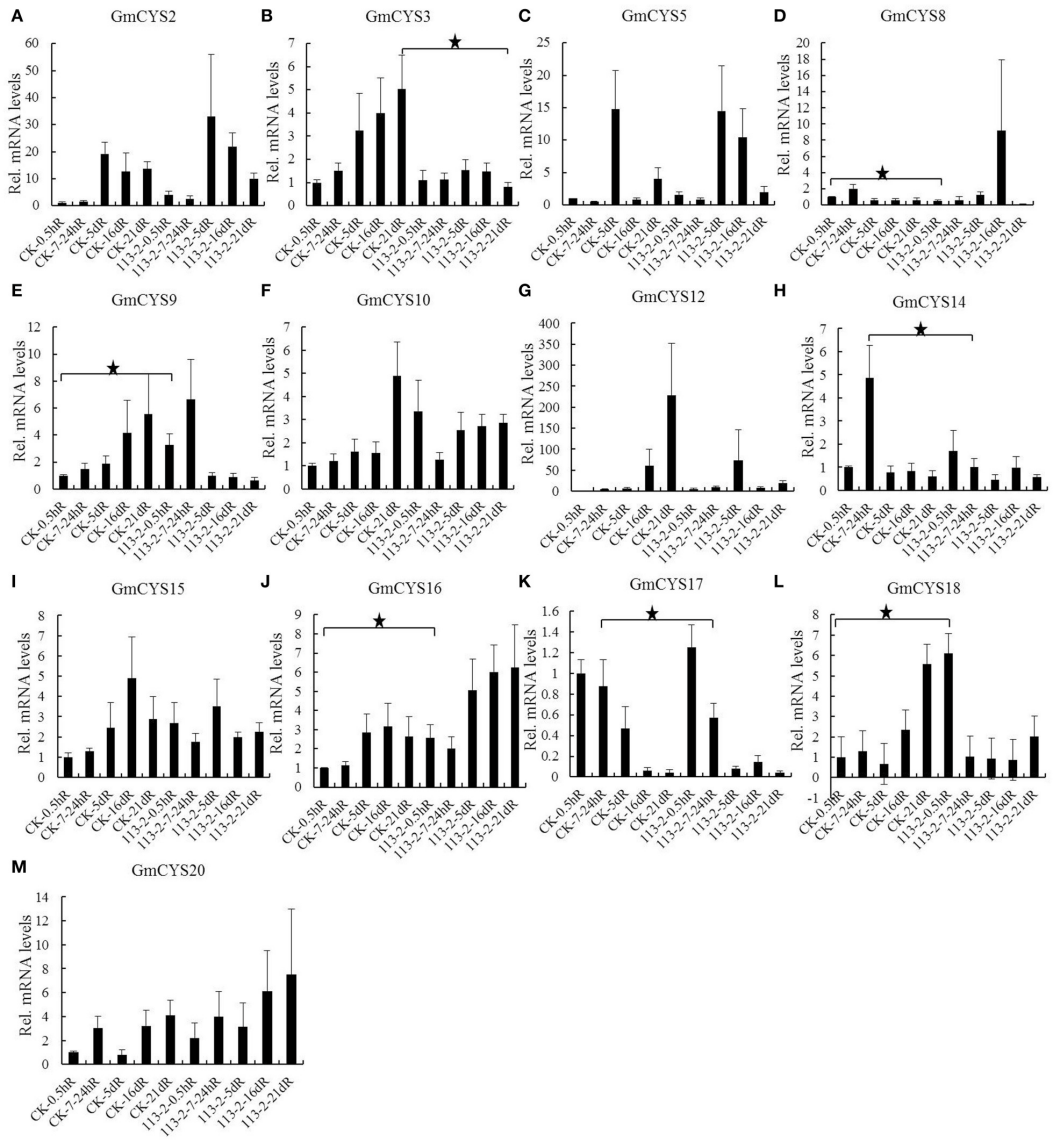
**Expression profile of cystatins family genes in soybean roots with and without inoculation of ***B. japonicum*** strain ***113-2*** at five post-inoculation time points**. Three biological replica samples were used to detect the expression levels of 13 cystatins. Relative expression levels of each cystatin gene were obtained by qPCR and normalized to the average expression level of soybean reference gene QACT, and calculated using the expression level in CK-0.5 hR as control. Student's *t*-test was done using software SPSS Statistics 17.0 and data are presented as the mean ± SE of 9 or 10 independent replicate experiments. “★” indicates statistical difference between the inoculated roots and CK roots (*t*-test, *p* < 0.05). **(A)** GmCYS2. **(B)** GmCYS3. **(C)** GmCYS5. **(D)** GmCYS8. **(E)** GmCYS9. **(F)** GmCYS10. **(G)** GmCYS12. **(H)** GmCYS14. **(I)** GmCYS15. **(J)** GmCYS16. **(K)** GmCYS17. **(L)** GmCYS18. **(M)** GmCYS20.

To determine whether cystatins play roles in symbiotic signal transduction, nodulation and early nodule development, 14 soybean cystatins that were expressed in root, root hairs and/or root at symbiotic condition were analyzed, and their expression levels in roots (CK and inoculated with *B. japonicum* strain 113-2) at 0.5 h, 7–24 h, 5, 16, and 21 d of post-inoculation were compared using *t*-tests to show statistical difference between CK and inoculated roots. Because no specific sequence was suitable for *GmCYS13* qPCR (Figure S1), only 13 soybean cystatins were detected (Figure 4). Inoculation of *B. japonicum* strain 113-2 significantly increased the expression of *GmCYS2, GmCYS9, GmCYS10, GmCYS12, GmCYS15, GmCYS16, GmCYS18*, and *GmCYS20* (>2 fold) at 0.5 hR of post-inoculation (Figures 4A,E,F,G,I,J,L,M), but significantly decreased the expression of *GmCYS8* (>2 fold) at 0.5 hR of post-inoculation (Figure 4D). The expression levels of *GmCYS8* and *GmCYS14* decreased (>2 fold) at 7–24 hR of post-inoculation (Figures 4D,H), while the expression level of *GmCYS9* increased (>2 fold) at 7–24 hR of post-inoculation (Figure 4E). The expression levels of *GmCYS17* decreased (>2 fold) at 5 dR of post-inoculation (Figure 4K), while the expression level of *GmCYS20* increased (>2 fold) at 5 dR of post-inoculation (Figure 4M). The expression of *GmCYS5* and *GmCYS8* in inoculated roots were increased (>2 fold) at 16 dR of post-inoculation, but decreased (>2 fold) at 21 dR of post-inoculation (Figures 4C,D), while for other 5 soybean cystatins (*GmCYS3, GmCYS9, GmCYS12, GmCYS15*, and *GmCYS18*), the expression levels were decreased (>2 fold) at 16 dR and/or 21 dR of post-inoculation (Figures 4B,E,G,I,L).

**Figure 5:**
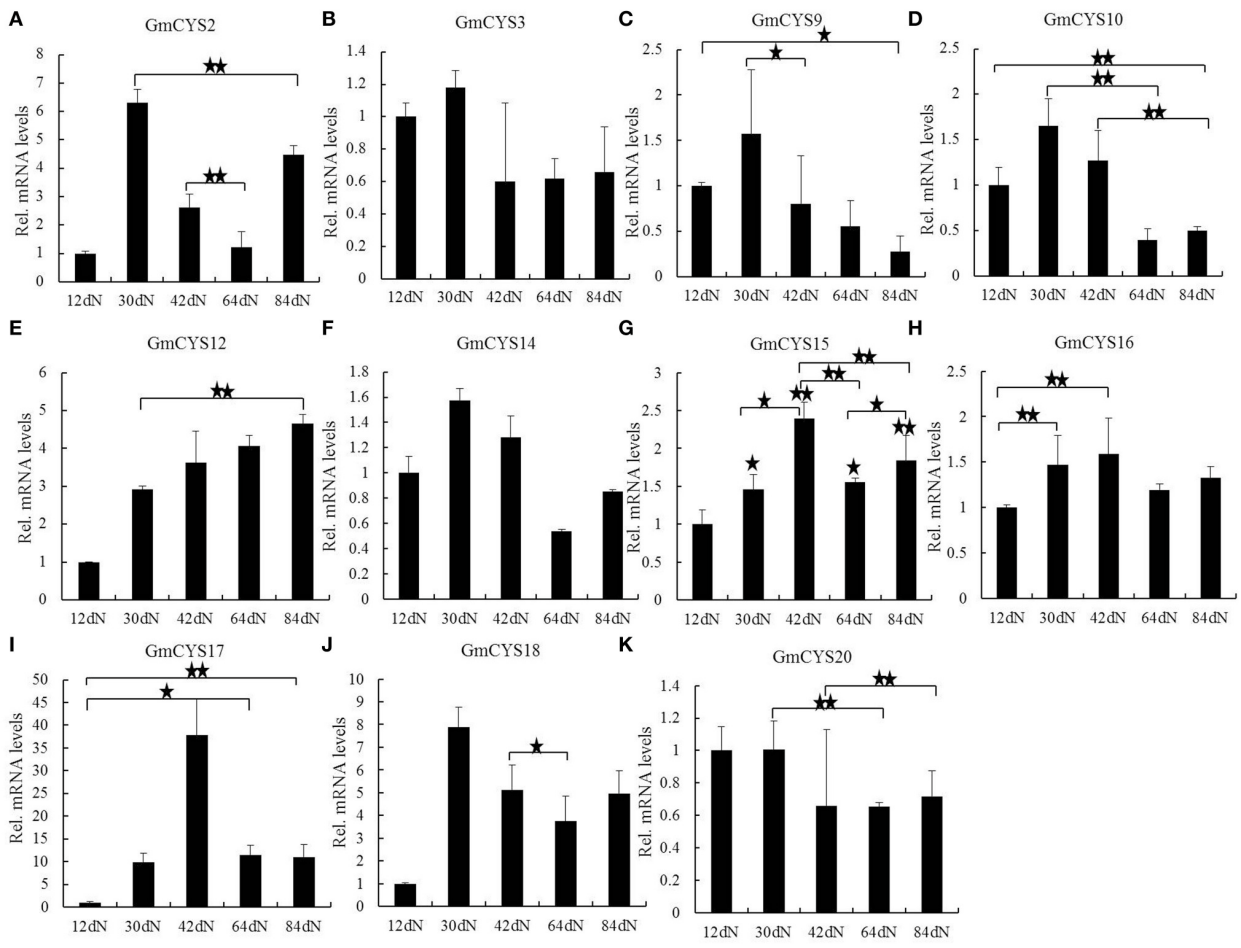
**Expression profile of cystatins family genes in soybean nodules at five important nodule development time points**. Three biological replica samples and three replicate reactions per sample were used to calculate the expression level of each cystatin during the nodule development. Relative expression levels of each cystatin gene were obtained by qPCR and normalized to the average expression level of soybean reference gene QACT, and calculated using the expression level in 12 dN as a starting point control. Data represent the mean ± SE of nine independent replicate experiments. “★”and “★★” indicate statistical difference between different nodule development time points (*t*-test, *p* < 0.05 or *p* < 0.01, respectively). “★”and “★★” without wire frame marker in **(G)**, indicate statistical difference compared to the 12 dN (starting point control). **(A)** GmCYS2. **(B)** GmCYS3. **(C)** GmCYS9. **(D)** GmCYS10. **(E)** GmCYS12. **(F)** GmCYS14. **(G)** GmCYS15. **(H)** GmCYS16. **(I)** GmCYS17. **(J)** GmCYS18. **(K)** GmCYS20.

Previous studies showed that some members of soybean cystatins are actively transcribed during nodule development and senescence (van Wyk et al., 2014). To determine which soybean cystatins play roles in nodule development and/or senescence, soybean cystatins that were expressed in nodule and/or nodules at symbiotic condition were analyzed using *t*-tests to show statistical difference between different nodule development time points. The results showed that 11 soybean cystatins (*GmCYS2, GmCYS3, GmCYS9, GmCYS10, GmCYS12, GmCYS14-18*, and *GmCYS20*) were differentially expressed in the nodules at five important nodule development time points (Figure 5). Among them, 4 soybean cystatins (*GmCYS3, GmCYS14, GmCYS16*, and *GmCYS20*) showed <2-fold expression variation in different nodules, suggesting that they were not regulated at the transcription level during soybean nodule development (Figures 5B,F,H,K). *GmCYS12, GmCYS17*, and *GmCYS18* were upregulated (>2 fold) between 12 dN and others (Figures 5E,I,J), *GmCYS2* was up-regulated (>2 fold) between 12 dN and other three nodules (30, 42, and 84 dN) and between 60 and 84 dN (Figure 5A), indicating that it may play a role in nodule senescence. The expressions of *GmCYS9* and *GmCYS10* were reduced (most were <2 fold) during the nodule development (Figures 5C,D). The expression levels of *GmCYS15* and *GmCYS17* reached their peaks at 42 dN (the nitrogen-fixation rate are relatively high; Figures 5G,I), suggesting that they may be regulated at the transcription level in the nitrogen-fixation process. The expression analysis results of 4 soybean cystatins (*GmCYS9, GmCYS10, GmCYS12*, and *GmCYS15*) agreed with transcriptional profile data (van Wyk et al., 2014). *GmCYS2* was downregulated between 30 and 64 dN (Figure 5A) and *GmCYS16* and *GmCYS20* showed no change at the transcription level during nodule development and senescence (Figures 5H,K).

### Expression analysis of soybean cystatin genes in response to ABA treatment in symbiosis with *B. japonicum* strain 113-2

**Figure 6:**
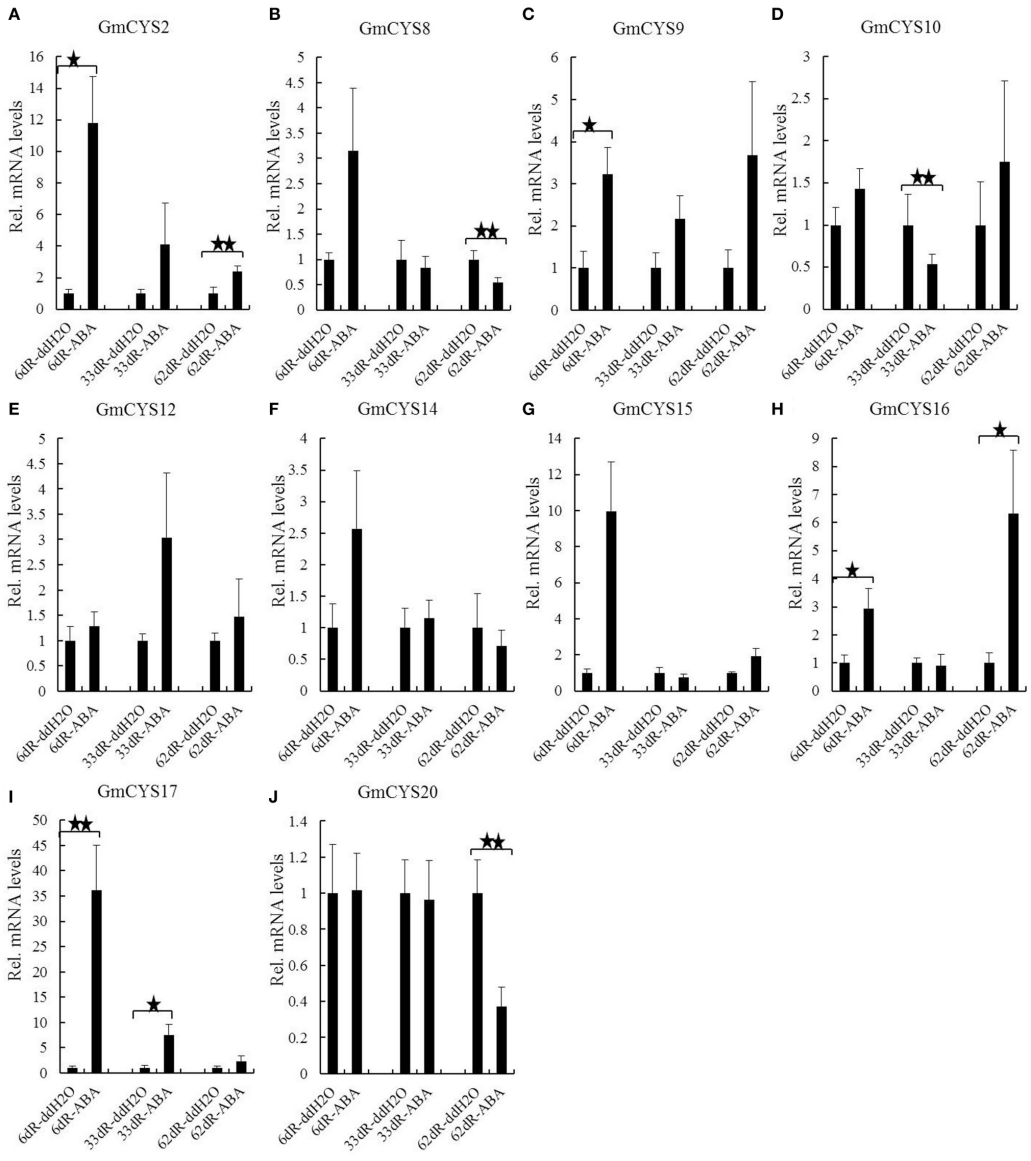
**Expression profile of soybean cystatin genes in response to ABA treatment in symbiosis with ***B. japonicum*** strain 113-2**. Three biological replica samples and three replicate reactions per sample were used to calculate the expression level of 10 cystatins in inoculated roots (with nodules, ddH_2_O, and ABA treatment) at 6, 33, and 62 days of post inoculation. Relative expression levels of each cystatin gene were obtained by qPCR and normalized to the average expression level of soybean reference gene QACT, and calculated using the expression level in ddH_2_O treatment samples as controls. Data are presented as mean ± SE of nine independent replicate experiments. “★”and “★★” indicate statistical difference between different treatment inoculated roots (*t*-test, *p* < 0.05 or p < 0.01, respectively). **(A)** GmCYS2. **(B)** GmCYS8. **(C)** GmCYS9. **(D)** GmCYS10. **(E)** GmCYS12. **(F)** GmCYS14. **(G)** GmCYS15. **(H)** GmCYS16. **(I)** GmCYS17. **(J)** GmCYS20.

As Table 2 described, 80% soybean cystatin genes have abscisic acid responsive elements (ABRE or CE3), suggesting that their expressions are induced by ABA, a negative regulator of symbiotic nitrogen fixation (Tominaga et al., 2009, 2010) that decreases nodule number (Phillips, 1971; Suzuki et al., 2004; Ding et al., 2008; Biswas et al., 2009) and negatively regulates nitrogen fixation (González et al., 2001). To confirm the response of soybean cystatins to ABA in symbiosis, the transcriptional levels of 10 soybean cystatins that contain ABRE and/or CE3 elements, and are expressed in inoculated roots (Tables 2, 3) at 6, 33, and 62 d of post-inoculation were compared using *t*-tests to show statistical difference among different treatment groups (with nodules, ddH_2_O and ABA treatment) (Figure 6). Three soybean cystatins (*GmCYS2, GmCYS9* and *GmCYS17*) were upregulated (>2 fold) after ABA treatment in inoculated roots at 6, 33, and 62 d of post-inoculation (Figures 6A,C,I). The expression of *GmCYS16* was increased (>2 fold) after ABA treatment in inoculated roots at 6 and 62 d of post-inoculation (Figure 6H), the expression level of *GmCYS8, GmCYS14* and *GmCYS15* was increased (>2 fold) after ABA treatment in inoculated roots at 6 d of post-inoculation (Figures 6B,F,G), and the expression level of *GmCYS12* was increased (>2 fold) after ABA treatment in inoculated roots at 33 d of post-inoculation (Figure 6E). The expression of *GmCYS10* was not changes (<2 fold) at the transcription level in soybean inoculated roots after ABA treatment (Figure 6D), suggesting that its expressions were not induced by ABA in soybean symbiosis with *B. japonicum* strain 113-2. Whether its expressions were induced by ABA in non-inoculated roots and/or other tissues need to be further explored.

### Functional analysis of a candidate gene *Glyma.15G227500* (*GmCYS16*) in nodulation

**Figure 7:**
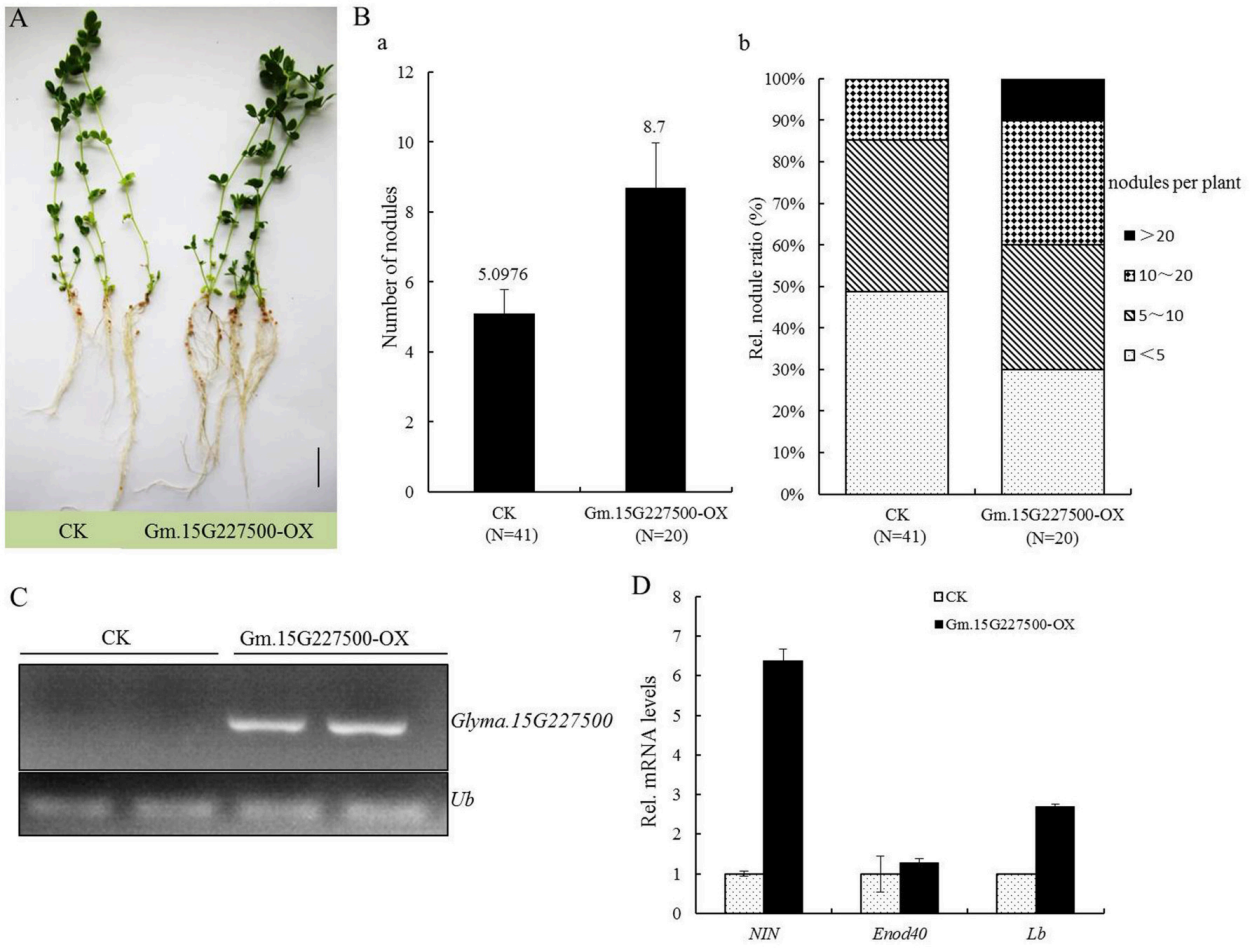
**Effect of ***Glyma.15G227500*** overexpression on symbiosis in ***L. japonicus***. (A)** Symbiotic phenotypes of transgenic plants at 32 days of post inoculation with *M. loti*. MAFF303099. Hairy roots expressing vector pU1301 served as a control. Independent transgenic plants were chosen for photography. Bars = 5 mm. **(B)** Nodule numbers of transgenic hairy roots with altered *Glyma.15G227500* transcript levels. Transgenic hairy roots expressing vector (pU1301) served as control. **(A)** Mean numbers of nodules per plant with standard deviation (SD) of *L. japonicus* expressing pMUb: Glyma.15G227500 (Gm.15G227500-OX) or the empty vector pU1301 (CK) at 32 days of post inoculation with *M. loti*. **(B)** Plants were divided into different groups on the basis of nodules per plant, and the relative ratios of various groups were calculated. **(C)** Semi-quantitative RT-PCR analysis of the transcript levels of *Glyma.15G227500* in the control and Gm.15G227500-OX hairy roots. **(D)** qPCR analysis of the transcript levels of *NIN, Enod40*, and *Lb* in the control and Gm.15G227500-OX hairy roots. Total RNA isolated from root system was used for qPCR. Relative expression levels of *NIN, Enod40*, and *Lb* transcripts in Gm.15G227500-OX hairy roots were calculated with reference to that of the control hairy roots.

As described above, when inoculated with *B. japonicum* strain 113-2, the expression of *GmCYS16* in soybean roots was significantly increased (Figure 4J), suggesting that *GmCYS16* may plays a role in nodulation. To confirm this result, *Glyma.15G227500* (*GmCYS16*) was expressed under the control of the maize (*Zea mays*) ubiquitin promoter (Glyma.15G227500-OX) in transgenic hairy roots of *L. japonicus* (Kumagai and Kouchi, 2003; Yuan et al., 2012). The phenotypes of nodulation were scored at 32 days of post-inoculation with *M. loti* MAFF303099, which expresses β-galactosidase (lacZ) as a constitutive marker for the presence of rhizobial cells (Tansengco et al., 2003). As shown in Figures 7A,B, more nodules were produced in Glyma.15G227500-OX hairy roots. The nodule number per root system was increased from 5.0976 in the control to 8.7 in Glyma.15G227500-OX hairy roots (Figure 7Ba). Analysis of the distribution of nodule number per plant revealed that 40% of Glyma.15G227500-OX hairy roots developed more than 10 nodules. In contrast, only 15% of the control hairy roots produced more than 10 nodules, and no hairy roots produced more than 20 nodules (Figure 7Bb). Semi-quantitative RT-PCR results showed that the expression level of *Glyma.15G227500* was abundant in Glyma.15G227500-OX hairy roots (Figure 7C). qPCR analyses of expressions of *NIN* and *ENOD40*, two early nodulin genes implicated in the processes of rhizobial entrance, nodule initiation, and subsequent organogenesis (Schauser et al., 1999; Kumagai et al., 2006), as well as *Lb* (leghemoglobin), a typical nodulin gene required for nitrogen fixation (Ott et al., 2005) showed that the expression levels of these three nodulin genes, especially *NIN* and *Lb*, were increased in Glyma.15G227500-OX hairy roots, as compared with those in the control hairy roots, (Figure 7D). These results suggest that Glyma.15G227500 plays a positive role in nodulation of *L. japonicus*.

## Discussion

Nodulation, nodule development and senescence directly affect nitrogen fixation efficiency. Previous studies have shown that inhibition of some cysteine proteases delays nodule senescence (Sheokand et al., 2005; Li et al., 2008). Thus, their nature inhibitors, cystatins, are very important in nodule development and senescence. Soybean is an important oil crop, food and feed material, providing large amounts of dietary proteins and edible oil and an excellent model for studying nitrogen fixation and nodule development. The inhibitory activities of 20 soybean cystatins including seven that were actively transcribed in nodules were analyzed (van Wyk et al., 2014). However, the exact roles of these cystatin genes in nodulation, nodule development, and senescence remain poorly characterized. To search for nodulation and nodule development-related cystatin genes in soybean, the detail information of genome-wide identification and characterization of 20 soybean cystatins were shown in this study to explore their functional diversities. Our study for the first time conducted the genome-wide survey of soybean cystatin family genes in nodulation and nodule development, and the resultant expression patterns of soybean cystatin family genes in symbiosis provide insights into their putative roles in nodulation and nodule development.

### Potential roles of soybean cystatin family genes in nodulation, nodule development, and senescence

Previous studies showed that cystatins could regulate nodulation and nodule development (Quain et al., 2015) and may play a role in regulating proteolysis in the process of nodules senescence and programmed cell death (van Wyk et al., 2014). However, which soybean cystatins play roles in nodulation, nodule development, and senescence is still largely unknown. Therefore, the expression profiles of soybean cystatins in five tissues (nodules. symbiotic condition, root. symbiotic condition, root, root hairs, and nodules) were comprehensively analyzed (Table 3) in present work, respectively (Figures 4, 5). The results showed that the expression levels of three soybean cystatins (*GmCYS2, GmCYS16*, and *GmCYS20*) were significantly increased in inoculated roots (Figures 4A,J,M), suggesting that they may play roles in nodulation. *GmCYS15* and *GmCYS17* showed highest expression at the stage with relatively high nitrogen-fixation rate (42 dN), indicating that they may play roles in the nitrogen-fixation process. For nodule senescence, previous transcriptional profile data has showed that expression of *GmCYS2, GmCYS15*, and *GmCYS16* were particularly increased during the onset of senescence (van Wyk et al., 2014). In this study, expression of *GmCYS15* and *GmCYS16* barely changed between 60 and 84 dN or between 42 and 84 dN (Figures 5G,H). Besides, the expression levels of *GmCYS12* and *GmCYS18* were significantly increased in roots at 0.5 h of post inoculation and during nodule developments (Figures 4G,L, 5E,J), suggesting that these two cystatins may play roles in nodulation and nodule development. These data indicated that these 7 soybean cystatin proteins (*GmCYS2, GmCYS12, GmCYS15, GmCYS16, GmCYS17, GmCYS18*, and *GmCYS20*) might play different roles in soybean nodule symbiosis. Their specific regulatory roles and distinct functions are interesting and worthy to be explored in the future.

### Characteristics of soybean nodulation and nodule development-related cystatin genes

As above described in the present work, the identified 20 soybean cystatin genes encode peptides with 97–245 amino acid residues, and different isoelectric points (pI) and structure characteristics (Table 1, Figures 1, 2). Three typical motifs for cystatin proteins, “QxVxG,” “G” in N-terminal and “W” residue were detected in most cystatins with only one exception (GmCYS4), and the other conserved “LARFAV” motif varied among the four Clades of soybean cystatin genes (Figure 1B). Five soybean cystatins (GmCYS10, GmCYS13, GmCYS14, GmCYS16, and GmCYS18) showed significant variations with the other 15 cystatins in their predicted three-dimensional structures (Figure 2), mainly due to these soybean cystatins have relatively complicated motifs and longer coding sequences than the others (except GmCYS18). Among them, 7 nodulation and nodule development-related cystatin proteins fall into different Clades (Figure 1) and have different structures (Table 1, Figure 2), indicating that there are no special clade cystatin protein or special structure in nodulation and nodule development-related cystatins.

Previous reports demonstrated that cystatin could inhibit activity of cysteine proteases in the process of plant seed germination (Martinez et al., 2009) and host immunity (Koiwa et al., 2000; van der Linde et al., 2012), and also play a key role in hypersensitive cell death and the regulation of defense against pathogens and insects (Bobek and Levine, 1992; Belenghi et al., 2003; Gholizadeh et al., 2005; Pogány et al., 2015). Besides, their expression is also responsive to abiotic stresses and hormone signals (Zhang et al., 2008; Wang et al., 2015). Similar to this study, various putative plant regulatory elements in response to different abiotic/biotic stresses (various environmental stresses) and hormone signals were shown in 3000 bp putative promoter fragments upstream of the 20 soybean cystatins (Table 2). The 7 nodulation and nodule development-related cystatin proteins have inconsistent plant regulatory elements in their promoters (Table 2). For hormones responsive elements, *GmCYS2* has elements in response to 5 different hormones, including abscisic acid, gibberellin acid, salicylic acid, auxin and jasmonic acid, *GmCYS12, GmCYS15, GmCYS16*, and *GmCYS17* have no auxin responsive elements, *GmCYS15* has no jasmonic acid responsive element, *GmCYS18* has no abscisic acid and gibberellin acid responsive elements, and *GmCYS20* has no gibberellin acid responsive elements. For environmental responsive elements, *GmCYS15, GmCYS16*, and *GmCYS18* have no MBS element, *GmCYS12* has no TC-rich repeats element, and *GmCYS15* has no circadian-regulated elements. Besides, *GmCYS2, GmCYS15, GmCYS17*, and *GmCYS18* have fungal elicitor responsive element, *GmCYS15* and *GmCYS17* have wound-responsive element, and *GmCYS15, GmCYS17*, and *GmCYS20* have ethylene-responsive element. Interesting, *GmCYS2* has a nodule specific factor binding site element (Nodule-site2, Table 2), which also exists in the promoter fragment of soybean leghemoglobin (*LBC3*), a typical nodulin gene required for nitrogen fixation (She et al., 1993). However, whether *GmCYS2* plays an important role in the soybean nitrogen fixation needs to be further studied. These results indicated that these 7 soybean cystatin proteins are not root nodule symbiosis (RNS)—specific cystatins, and have different putative biological functions.

### A special branch for legume plant cystatins containing only one soybean cystatin related to nodulation and nodule development

As described in the evolutionary relationships among the cystatin family in rice, barley, *A. thaliana, N. tabacum* and four legume plants, Group A cystatins are special branch for legume plant, Group B cystatins only contain non-legume plant cystatins (non-legume plant dicotyledon and monocotyledon), and Group C cystatins are both legume plants and non-legume plant related (Figure 3). Of the 7 cystatins related to nodulation and nodule development, only *GmCYS15* fall into Group A, the other 6 cystatins fall into Group C (Figure 3). These results indicated that cystatins in the special branch are not special for legume property-nodulation and nitrogen fixation and may possess other biological functions.

In summary, 20 cystatin family genes were identified in soybean genome through a genome-wide survey with the aim to search for nodulation and nodule development-related cystatin genes in soybean. Characterization of their gene sequences, structures, and promoter sequences as well as phylogenetic analysis revealed special characteristics of cystatin family genes in soybean. Expression profiling of the cystatin genes in soybean symbiosis with *B. japonicum* 113-2 indicated that 7 cystatin family genes might play different roles in nodulation and nodule development. The functional analysis showed that the candidate gene *Glyma.15G227500* (*GmCYS16*) likely to play a positive role in soybean nodulation. These investigations and analyses shed new light on the research of the functional diversity of all members in the soybean cystatin family proteins, and could increase our knowledge on their functions in regulating soybean nodulation and nodule development, and provide helpful clues for investigation of nodule-specific cysteine proteases in soybean.

## Author contributions

SY and XZho designed this work. SY wrote the manuscript. SY and RL performed most of the experiments. HC, CZ, LC, QH, ZS, XZha, SC, ZY, DQ, and LW contributed substantially to the completion of this work. All authors read and approved the final manuscript.

### Conflict of interest statement

The authors declare that the research was conducted in the absence of any commercial or financial relationships that could be construed as a potential conflict of interest.
