# Supplementary material for: Search for Nodulation and Nodule Development-Related Cystatin Genes in the Genome of Soybean (Glycine max)
Source: Front Plant Sci. 2016 Oct 25;7:1595. doi: 10.3389/fpls.2016.01595 (PMC5078837; doi:10.3389/fpls.2016.01595)
Supplement: Supplementary file 1 [file Table1.DOCX]

**Table S1: GenBank accession numbers of Cystatin genes in seven plant species.**

|  | | **GenBank accession numbers** | | | |  |
| --- | --- | --- | --- | --- | --- | --- |
| ***Medicago trunctula*** | *MtCYS1*(Medtr0013s0060) | | *MtCYS2*(Medtr0013s0070) | *MtCYS3*(Medtr0219s0010) | *MtCYS4*(Medtr0437s0020) | |
|  | *MtCYS5*(Medtr2g026040) | | *MtCYS6*(Medtr2g028080) | *MtCYS7*(Medtr2g028520) | *MtCYS8*(Medtr2g076740) | |
|  | *MtCYS9*(Medtr2g076800) | | *MtCYS10*(Medtr2g103740) | *MtCYS11*(Medtr3g043750) | *MtCYS12*(Medtr3g084780) | |
|  | *MtCYS13*(Medtr3g451210) | | *MtCYS14*(Medtr3g452280) | *MtCYS15*(Medtr4g017620) | *MtCYS16*(Medtr4g040310) | |
|  | *MtCYS17*(Medtr4g095630) | | *MtCYS18*(Medtr4g133540) | *MtCYS19*(Medtr5g088770) | *MtCYS20*(Medtr5g091260) | |
|  | *MtCYS21*(Medtr6g022430) | | *MtCYS22*(Medtr6g078440) | *MtCYS23*(Medtr8g012950) | *MtCYS24*(Medtr8g041410) | |
|  | *MtCYS25*(Medtr8g079410) | | *MtCYS26*(Medtr8g080920) | *MtCYS27*(Medtr8g098380) | *MtCYS28*(Medtr5g091280) | |
|  | *MtCYS29*(Medtr5g091330) | | *MtCYS30*(Medtr5g091360) | *MtCYS31*(Medtr5g091520) | *MtCYS32*(Medtr5g098550) | |
|  | *MtCYS33*(Medtr5g098560) | | *MtCYS34*(Medtr6g022420) |  |  | |
|  |  | |  |  |  | |
| ***Lotus japonicus*** | *LjCYS1*(Lj0g3v0063219) | | *LjCYS2*(Lj1g3v4081850) | *LjCYS3*(Lj2g3v0126210) | *LjCYS4*(Lj4g3v2575030) | |
|  | *LjCYS5*(Lj4g3v3110650) | | *LjCYS6*(Lj6g3v1008020) | *LjCYS7*(Lj6g3v1753280) |  | |
|  |  | |  |  |  | |
| ***Common bean*** | *PvCYS1*(Phvul.001G264400) | | *PvCYS2*(Phvul.003G065700) | *PvCYS3*(Phvul.003G066000) | *PvCYS4*(Phvul.003G066200) | |
|  | *PvCYS5*(Phvul.003G066500) | | *PvCYS6*(Phvul.003G066600) | *PvCYS7*(Phvul.003G084300) | *PvCYS8*(Phvul.006G018600) | |
|  | *PvCYS9*(Phvul.006G142600) | | *PvCYS10*(Phvul.011G196600) |  |  | |
|  |  | |  |  |  | |
| ***Hordeum vulgare*** | *HvCYS1*(Y12068) | | *HvCYS2*(AJ748337) | *HvCYS3*(AJ748338) | *HvCYS4*(AJ748344) | |
|  | *HvCYS5*(AJ748340) | | *HvCYS6*(AJ748341) | *HvCYS7*(AJ748345) | *HvCYS8*(AJ748343) | |
|  | *HvCYS9*(AJ748339) | | *HvCYS10*(AJ748342) | *HvCYS11*(AJ748346) | *HvCYS12*(AJ748347) | |
|  | *HvCYS13*(AJ748348) | |  |  |  | |
|  |  | |  |  |  | |
| ***Oryza sativa*** | *OsCYS1*(Os01g16430) | | *OsCYS2*(Os01g58890) | *OsCYS3*(Os01g68660) | *OsCYS4*(Os01g68670) | |
|  | *OsCYS5*(Os03g07610) | | *OsCYS6*(Os03g07620) | *OsCYS7*(Os03g07650) | *OsCYS8*(Os03g07660) | |
|  | *OsCYS9*(Os03g07700) | | *OsCYS10*(Os03g11160) | *OsCYS11*(Os03g11170) | *OsCYS12*(Os03g11180) | |
|  | *OsCYS13*(Os03g31510) | | *OsCYS14*(Os04g28250) | *OsCYS15*(Os05g33880) | *OsCYS16*(Os05g41460) | |
|  | *OsCYS17*(Os09g08100) | | *OsCYS18*(Os10g26100) |  |  | |
|  |  | |  |  |  | |
| ***Arabidopsis thaliana*** | *AtCYS1*(AT2G31980) | | *AtCYS2*(AT2G40880) | *AtCYS3*(AT3G12490) | *AtCYS4*(AT4G16500) | |
|  | *AtCYS5*(AT5G05110) | | *AtCYS6*(AT5G12140) | *AtCYS7*(AT5G47550) |  | |
|  |  | |  |  |  | |
| ***Nicotiana tabacum*** | *NtCYS1*(KF113570) | | *NtCYS2* (KJ725113) | *NtCYS3* (KJ725114) | *NtCYS4* (KJ725115) | |
|  | *NtCYS5* (KJ725116) | | *NtCYS6* (KJ725117) | *NtCYS7* (KJ725118) | *NtCYS8* (KJ725119) | |
|  | *NtCYS9* (KJ725120) | | *NtCYS10* (KJ725121) |  |  | |
