# Supplementary material for: Search for Nodulation and Nodule Development-Related Cystatin Genes in the Genome of Soybean (Glycine max)
Source: Front Plant Sci. 2016 Oct 25;7:1595. doi: 10.3389/fpls.2016.01595 (PMC5078837; doi:10.3389/fpls.2016.01595)
Supplement: Supplementary file 3 [file Image1.PDF]

```
GmCYS13-3-UTR   ................................................................................     0
GmCYS14-3-UTR   TCTTTATAAGGTATAAAATACGCTTTTAATCTCTGTGCTCTCAATAAATATTAGTATTAGTTTTCATATTTTTATTTTGA    80
Consensus

GmCYS13-3-UTR   ................................................................................     0
GmCYS14-3-UTR   TCAAATTAATCTTTAAACCTCAGAAAAATAACAATGGTTTCATCATTACTTTTATTGTGAGCGAGGAGGAACACATTATT   160
Consensus

GmCYS13-3-UTR   ................................................................................     0
GmCYS14-3-UTR   ATCTTTTTTTTTAATTCAAGAATTAAATTAATTAAAACTAAAATTTGAAAACTAAAATTAAGTTTTATTCTTTTACTAAA   240
Consensus

GmCYS13-3-UTR   ................................................................................     0
GmCYS14-3-UTR   CTACCATCACCAAATATTCTGGATATTCATATCCATAGTGTCGTTCTCTGTTGGAATTATTAAATGTGTTTTGTCTTTCT   320
Consensus

GmCYS13-3-UTR   ....TCTTTATAAGGTTACAGAAATAAATGGTCGCAAGGTGAAAGTTGTACTAAAATTTATTTTTTATAAAAAAAATCGA    76
GmCYS14-3-UTR   CGTTTTTTTTTCAGGTTACAGAAATAAATGGTCGCAAGCTGAAAGTTGTACTAAAATTTATTTTTTATAAAAAAA.TCGA   399
Consensus           t ttt t aggttacagaaataaatggtcgcaag tgaaagttgtactaaaatttattttttataaaaaaa tcga

GmCYS13-3-UTR   AGGTAGTATTAAATATGTTATATGTATGTATTGTGCAGAATAAATGCAGCCACGTACTATATATAATGGTACATGCGGTG   156
GmCYS14-3-UTR   AGGTAGTATTAAATATGTTATATGTATGTATTGTGCGGAATAAATGCAGCCACGTACTATATATAATGGTACATACGGTG   479
Consensus       aggtagtattaaatatgttatatgtatgtattgtgc gaataaatgcagccacgtactatatataatggtacat cggtg

GmCYS13-3-UTR   TAGGGCTGTACAAAT.GGGCATCCTATTTCAATATTAACGACCACTAATAAATATTACCATTGGAGTTATTACGTATTGA   235
GmCYS14-3-UTR   TAGGGCTGTACAAGTTGGGCATCCTATTTCAATATTAACGACCACTAATAAATATTACCATTGGAGTTATTACGTATTGA   559
Consensus       tagggctgtacaa t gggcatcctatttcaatattaacgaccactaataaatattaccattggagttattacgtattga

GmCYS13-3-UTR   TTATTCTGTATTTACGCGTAAATGGCTAAATGTTTCCAATTGATGCACTAAGTGAAGCAAACAAGTGTTCCTTTGTTTAT   315
GmCYS14-3-UTR   TTATTCTGTATTTACGCGTAAATGGCTAAATGTTTCCAATTGATGCACTAAGTGATGCAAACAAATGTTCCTTTGTTTAT   639
Consensus       ttattctgtatttacgcgtaaatggctaaatgtttccaattgatgcactaagtga gcaaacaa tgttcctttgtttat

GmCYS13-3-UTR   TTTTTAAGGGCAAG.........                                                            329
GmCYS14-3-UTR   TTTTTAAGGGCAAGATGAAAAAA                                                            662
Consensus       tttttaagggcaag
```

Figure S1: Alignment of 3´ UTR sequences of GmCYS13 and GmCYS14.
